# The convoluted process of diagnosing pulmonary mycosis caused by *Exophiala dermatitidis*: a case report

**DOI:** 10.1186/s12879-022-07399-y

**Published:** 2022-05-04

**Authors:** Zhengtu Li, Jianli Tang, Jinping Zhu, Mingzhou Xie, Shaoqing Huang, Shaoqiang Li, Yangqing Zhan, Weiqi Zeng, Teng Xu, Feng Ye

**Affiliations:** 1 State Key Laboratory of Respiratory Disease, National Clinical Research Center for Respiratory Disease, National Center for Respiratory Medicine, Guangzhou Institute of Respiratory Health, The First Affiliated Hospital of Guangzhou Medical University, 151 Yanjiang Xi Road, Guangzhou, 510120 Guangdong China; 2 Department of Respiratory and Critical Care Medicine, Songgang People’s Hospital, Shenzhen, 518105 China; 3 Vision Medicals Co. Ltd., Guangzhou, 510663 China

**Keywords:** *Exophiala dermatitidis*, Tissue mNGS, Antifungal therapy, Follow-up, Case report

## Abstract

**Background:**

Etiological diagnosis is a key step in the treatment of patients with rare pulmonary mycosis, and the lack of understanding of this disease and lack of specific markers for the detection of rare species, such as *Exophiala dermatitidis,* add to the difficulty in diagnosing the condition. Therefore, improving the diagnostic strategies for this disease is very important.

**Case presentation:**

A 52-year-old man presented with cough, sputum production and hemoptysis; chest computed tomography (CT) revealed multiple bilateral lesions. The pathogen was unable to be identified after three biopsies. Subsequently, we performed combined tissue metagenomic next-generation sequencing (mNGS). The results of mNGS and a good therapeutic response helped to identify the causative pathogen as *Exophiala dermatitidis*. Finally, the patient was diagnosed with *Exophiala dermatitidis* pneumonia.

**Conclusions:**

Combining molecular techniques, such as mNGS, with clinical microbiological tests will improve the rate of positivity in the diagnosis of rare fungal infections, and the importance of follow-up should be emphasized.

**Supplementary Information:**

The online version contains supplementary material available at 10.1186/s12879-022-07399-y.

## Background

*Exophiala dermatitidis,* a rare fungal species, can cause pulmonary mycosis. Due to the lack of understanding of this condition as well as the lack of specific markers, diagnosing pulmonary mycosis caused by *Exophiala dermatitidis* is challenging. The key requirement for the management of the disease and the development of treatment strategies for infected patients is the identification of pathogens from clinical samples [1]. However, traditional methods for the detection of pathogenic microorganisms, including morphological detection, culture, microscopy, antigen–antibody detection, and nucleic acid detection, have inevitable shortcomings. For example, microbial culture is time consuming and has a low positivity rate [2], and molecular diagnostic methods, such as PCR, can detect one or more target-specific pathogens but miss pathogens that are not targeted by the primer set, making this approach unsuitable for the detection of pathogens in difficult-to-diagnose infectious diseases. A new technology, metagenomic next-generation sequencing (mNGS), is being widely used in clinical practice to detect bacteria, fungi, viruses, atypical pathogens, parasites, and new pathogens in respiratory specimens, overcoming the limitations of traditional microbial detection methods. Chen et al.[3] used this technique to sequence specimens from 93 lower respiratory tract infection (LRTI) patients, and this approach resulted in definite or probable pathogen detection in 65% of the specimens, while only 20% and 35% were positive by culture and other microbial detection methods, respectively.

Here, we report a rare case of pulmonary mycosis caused by *Exophiala dermatitidis* identified by mNGS, which ultimately resulted in an improved therapeutic effect.

## Case presentation

A 52-year-old man developed a dry cough after cold exposure one month prior but lacked symptoms such as fever, night sweats, and dyspnea. He responded poorly to treatment with traditional Chinese medicine in the local hospital and experienced cough exacerbation with the production of white sputum. He then visited a comprehensive Western medicine hospital, where a chest computed tomography (CT) scan showed multiple lesions and indications of possible fungal infection. A serum *Aspergillus* antigen test (GM test) was positive, but the results of other pathogen tests (including sputum smear and culture) were negative. The diagnosis was a fungal infection, and he was prescribed oral voriconazole (200 mg, bid). The symptoms of cough and sputum production improved. However, hemoptysis developed on the sixth day of treatment, and the patient visited our hospital (the First Affiliated Hospital of Guangzhou Medical University, Guangzhou, China).

He had a smoking index of 200 (20 cigarettes/day for 20 years), body mass index (BMI) of 19.47 kg/m^2^ and no specific underlying disease.

**Fig. 1:**
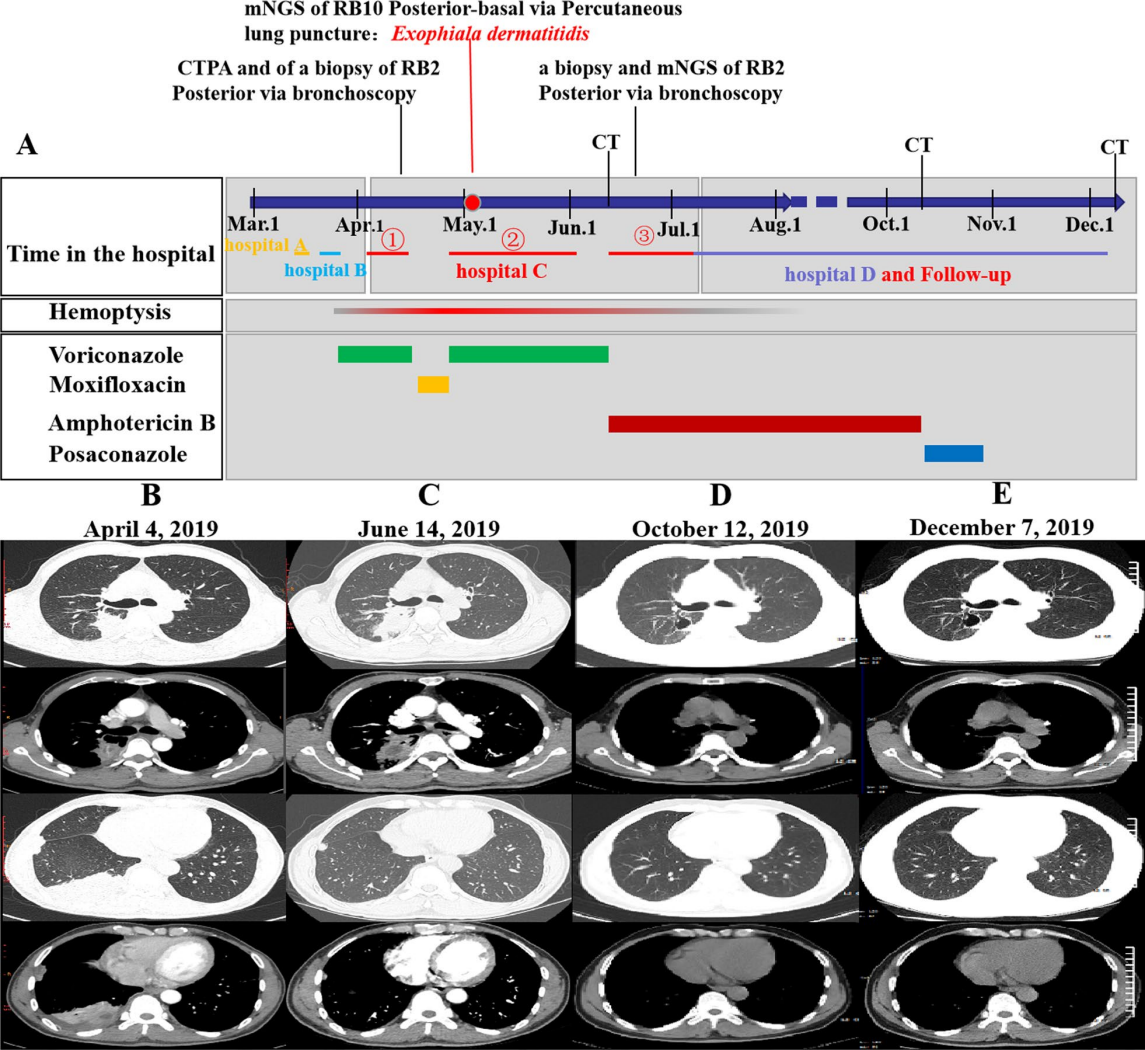
Timeline of clinical events and pharmacological treatments. The patient visited four hospitals successively; Hospital C was our hospital. He presented to our hospital three times and underwent three pathological biopsies and two mNGS tests. We performed follow-up chest CT scans and adjusted the medication accordingly. Hemoptysis resolved after treatment with amphotericin B. **A** Results of the first chest CT performed in our hospital (2019-4-11). Multiple nodules, mass lesions, and patchy shadows were present throughout both lungs, mainly under the pleura and particularly in the right upper and lower lobes. The results also showed unclear borders, an internal cavity, and moderate enhancement, with a standardized uptake value (SUV) of approximately 34/60/71 µ. **B** Results of chest CT re-examination after two months (2019-6-14). Some lesions in the posterior right upper and right lower lobes progressed, while others had been obviously absorbed. **C** Results of chest CT after 3 months of amphotericin B treatment (2019-10-12). The lesions had been obviously absorbed. **D** Results of follow-up chest CT after drug withdrawal for more than two months (2019-12-7). No change was observed compared to the last imaging findings

At admission, no significant abnormalities were found on physical examination. Chest CT revealed multiple nodules and mass lesions throughout both lungs, mainly under the pleura, particularly in the right upper and lower lung (Fig. 1B, April 4, 2019). Routine laboratory tests revealed a CD4 + /CD8 + ratio of 1.03 (1.4–2.0), absolute T lymphocyte count of 705/µL (955–2860), and absolute T helper lymphocyte count of 344/µL (550–1440). A white blood cell count of 9560/µL, with 7800/μL neutrophils, was also observed. The procalcitonin level was < 0.05 ng/mL. A blood T-SPOT tuberculosis (TB) test (interferon gamma release assay, IGRA**)** was positive (antigen A (ESAT-6): 21 spots), but sputum and bronchoalveolar lavage fluid (BALF) cultures, sputum TB DNA detection; BALF X-Pert galactomannan (GM), (1 → 3)-β-D-glucan (BG) assays; and the *Cryptococcus* antigen test was negative. Levels of lung tumor indicators were normal. Levels of antinuclear antibodies and vasculitis indicators were normal. Two successive biopsies were performed via bronchoscopy (posterior RB2) and percutaneous lung puncture guided by B-mode ultrasound (posterior-basal RB10). Unfortunately, the histopathologic results indicated inflammation with an unknown cause, and the antacid fluorescence and culture results were negative.

**Fig. 2:**
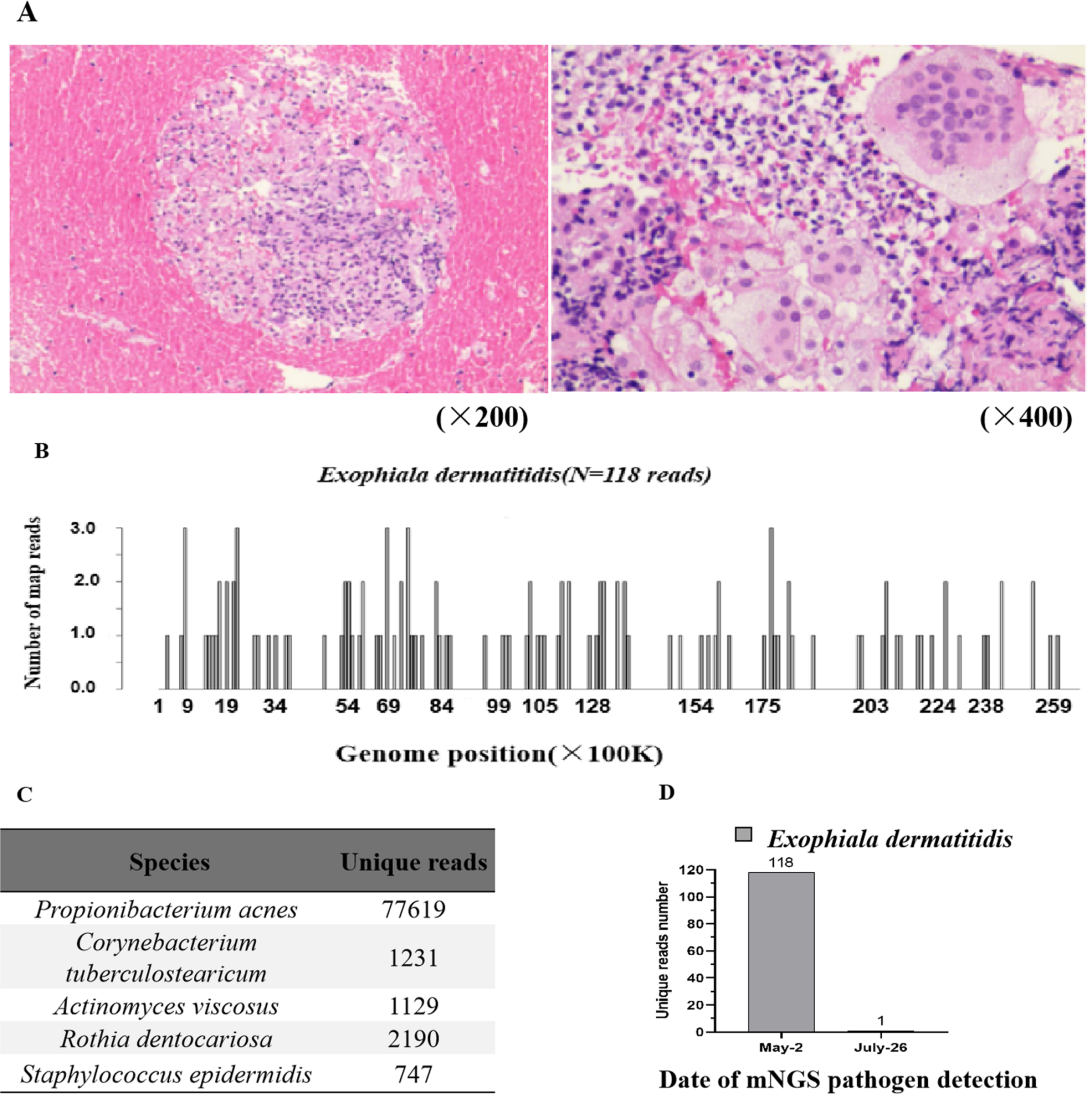
**A** Pathological results for posterior RB2. Granulomatous inflammation. Under the microscope, lymphocyte infiltration was noted, and individual multinucleated giant cells were observed. Special staining results: acid-fast 1 (−), acid-fast 2 ( +), Gram (−), Grocott methenamine silver (GMS) (−), periodic acid-Schiff (PAS) (−), acid-fast fluorescence ( +), fungal fluorescence (−). **B**, **C** Results of mNGS detection in specimens obtained by percutaneous lung puncture. These results show the specific fungal sequences of *Exophiala dermatitidis* and other suspected respiratory/skin flora. **D** Comparison of the number of mNGS reads detected in the two biopsy specimens

After stopping oral voriconazole because of a lack of evidence of fungal infection, the patient was discharged from the hospital, and his treatment was adjusted to oral moxifloxacin hydrochloride (0.4 g qd). Half a month later, he returned to our hospital due to hemoptysis recurrence. Based on this condition and other clinical features, we considered an infectious disease. From a paraffin-embedded tissue sample (from posterior-basal RB10), clinical mNGS identified 118 unique sequence reads for *Exophiala dermatitidis* (Fig. 2B) in addition to normal respiratory/skin flora[4, 5] (Fig. 2C). The close identity with two sequences from *Exophiala spp.* downloaded from the NCBI is shown in Additional file 1: Fig. S1.

Without other etiological evidence, we diagnosed the patient with *Exophiala dermatitidis* pneumonia. Because of the lack of drug susceptibility data, we had to review the literature and refer to our experience with medications. After diagnosis, we supposed that the previously administered course of voriconazole treatment was insufficient. Therefore, the patient was treated with intravenous voriconazole (the first dose was 300 mg (6 mg/kg) bid, followed by 200 mg (4 mg/kg) bid) for maintenance. The patient was switched to oral voriconazole (200 mg bid) after discharge.

During a telephone follow-up, the patient told us that his clinical symptoms had been significantly alleviated. Two months later, he was hospitalized for assessment and complained of occasional hemoptysis. Laboratory tests indicated liver dysfunction, with an alanine aminotransferase (ALT) level of 124.4 U/L and a γ-glutamyl transpeptidase level of 107.7 U/L. The trough serum concentration of voriconazole was 0.33 µg/mL (0.5–5.0). Re-examination with chest CT revealed the progression of some lesions in the posterior right upper lung and right lower lung, while some of the other lesions had been absorbed (Fig. 1C, June 14, 2019). Based on the adverse effects and poor efficacy of the drug, on June 22, 2019, we decided to stop voriconazole and adjust the treatment plan of amphotericin B (started at a dose of 5 mg/d and gradually increased until a maintenance dose of 30 mg/d was reached). Additionally, we strengthened hydration treatment for kidney protection and orally administered potassium to prevent hypokalemia. The patient had no further hemoptysis events and tolerated treatment well. When the cumulative dose of amphotericin B reached 555 mg, he returned to our hospital for imaging re-examination, and no increase in the size of the lesion was observed.

To clarify whether the lesion in the right upper lung was homogenous with the posterior-basal RB10 sample, we sampled posterior RB2 lung tissue again via bronchoscopy. Pathological examination revealed granulomatous inflammation and antacid 2 and antacid fluorescence positivity (Fig. 2A). The paraffin-embedded tissue was subjected to clinical mNGS again, and the results showed that only specific *Exophiala dermatitidis* sequence reads were detected, and the number of reads was reduced by only 1 (Fig. 2D). Based on the clinical characteristics, including lack of fever and night sweats; potential positivity on the blood T-SPOT test due to possible prior TB infection, which likely induced memory cell generation in the body; repeatedly negative BALF TB-DNA, X-Pert, sputum and BALF TB culture results; and the poor response to antifungal therapy in the early stage, we did not consider TB infection. We continued the current treatment regimen in a local hospital and performed follow-up. After 3 months, the cumulative dose of amphotericin reached 2935 mg, and chest CT showed that lung lesions had been obviously absorbed, leaving only a thin-walled cavity (Fig. 1D, October 12, 2019). The patient had no symptom of cough or hemoptysis. We discontinued amphotericin B treatment and administered posaconazole (200 mg, qid) orally to avoid prolonged intravenous drug use. After half a month, there was no change in the lesion. We judged that the patient had achieved clinical cure and terminated treatment. Two months later, a follow-up chest CT scan showed no change (Fig. 1E, December 7, 2019). Finally, we confirmed that the pathogen causing the patient's infection was *Exophiala dermatitidis*. The patient’s clinical timeline and schedule of medications are shown in Fig. 1-A.

## Discussion and conclusions

*Exophiala dermatitidis* is a saprophytic fungus that is usually present in decaying plants and soil. It can also be found in high-temperature and high-humidity areas, such as dishwashers, bathrooms or sauna facilities, in anthropogenic environments. It has also been isolated from railroad sleepers in subtropical areas. It is a rare conditional pathogen that often causes phaeohyphomycosis [6]. It can colonize lung transplantation wounds and cause rapidly progressing invasive dermatitis [7], which has been reported in cystic fibrosis (CF) patients. A prospective study by Lebecque et al. found that 5.8% of CF patients had at least one sputum culture positive for this common respiratory tract-colonizing fungus. This fungus is considered a potential marker for atypical CF in patients with chronic respiratory diseases and recurrent lung infections [8]. Central nervous system infections due to this fungus have also been reported in Asian populations with normal immunity [9].

In summary, in our case report, the patient had no clear underlying lung disease or immunosuppressive status; however, he was a middle-aged male with weight loss and long-term heavy smoking. Unfortunately, pulmonary function testing could not be performed because of multiple hemoptysis events, and it was impossible to assess whether there was chronic airway inflammation.

Although we did not obtain definitive evidence to confirm whether the patient was immunocompromised, T-cell subpopulation testing one month after illness onset showed that T-cell levels were decreased, and soon after treatment, related indices gradually returned to normal. Therefore, we speculated that he might have experienced a relatively significant reduction in CD4 cell levels in the early stage, which provided an opportunity for *Exophiala dermatitidis* infection.

A standardized diagnostic tool for the detection of *Exophiala spp.* is still lacking. Specific IgG detection tools have not yet been commercialized or widely applied. In addition, slow growth, large differences in morphological structures within the same species, and a lack of species-specific morphology all impose substantial challenges. Although matrix-assisted laser desorption ionization time-of-flight (MALDI-TOF) mass spectrometry has been proven to be the best method for the rapid identification of pathogenic yeast from cultures [6], the databases used in routine clinical practice may not include *Exophiala spp*. [7]. Currently, molecular methods are increasingly replacing morphological methods for the identification of fungi. Species-specific PCR was introduced by Nagano et al. in 2008 and was the first successful sequencing method, which is based on sequencing of the ribosomal DNA (rDNA) operon and its region [10]. Most researchers believe that sequencing based on rDNA transcription spacers can obtain accurate species identification [7, 11]. A revision and update of the consensus definitions of invasive fungal disease by the European Organization for Research and Treatment of Cancer and the Mycoses Study Group Education and Research Consortium (EORTC/MSGERC) (2019) added tissue nucleic acid diagnosis as a criterion for proven invasive fungal disease [12]. However, tissue nucleic acid diagnosis data is based mostly on *Aspergillus* infection, and its value in the diagnosis of other rare pulmonary fungal diseases needs additional research. In our case, multiple pathologies suggested inflammation, one of which suggested TB infection, but combined with the clinical characteristics, we were skeptical of TB infection and finally diagnosed the patient with *Exophiala dermatitidis* infection using tissue nucleic acid testing combined with treatment response data.

Regarding treatment, the European Society of Clinical Microbiology and Infectious Diseases (ESCMID)/European Confederation of Medical Mycology (ECMM) guidelines recommend the use of azoles, such as voriconazole and posaconazole. Several antifungal susceptibility test results for *Exophiala dermatitidis* have been reported. Nweze et al. analyzed 16 isolates and found that almost all strains were sensitive to amphotericin B, 5-fluorocytosine, itraconazole, fluconazole and voriconazole [13]. Another study showed that amphotericin B was active against all tested species except *Exophiala mesophila*, with minimum inhibitory concentration (MIC) values of 1 μg/mL [11]. Gao et al. reported that voriconazole, itraconazole and posaconazole were active against *Exophiala dermatitidis*[14]. Triazole combined with flucytosine, amphotericin B combined with flucytosine, amphotericin B combined with terbinafine, and posaconazole combined with amphotericin B all showed synergistic activity [15–18]. In contrast, echinocandins have weak antifungal activity. It is worth noting that a breakthrough infection during prevention and treatment with micafungin has also been reported [19].

After re-examining our case, we diagnosed *Exophiala dermatitidis* infection based on the mNGS results and continued the oral administration of voriconazole. After 2.5 months, most of the lesions had been significantly absorbed, but the dorsal lesion had progressed, and indications of drug-induced liver damage were observed. Considering a possible decrease in drug sensitivity, we adjusted the treatment to amphotericin B combined with posaconazole. Because of the cost involved, amphotericin B was finally administered. After 3 months, the lesion had been significantly absorbed. We adjusted the treatment to posaconazole for half a month in the later period. There was no evidence of recurrence at follow-up after treatment withdrawal. Although there is currently no established targeted PCR method for the detection of *Exophiala dermatitidis* from formalin-fixed paraffin-embedded (FFPE) tissue DNA, we believe a definitive diagnosis of pulmonary *Exophiala dermatitidis* infection can be established based on the current guidelines and during the treatment process.

Due to the limited culture technology available for the detection of this rare fungus, we failed to obtain drug sensitivity results; previous studies found that susceptibility test results for clinical isolates of *Exophiala dermatitidis* showed some differences. Therefore, if our patient had experienced a poor therapeutic effect, it would have been very difficult to select an appropriate antifungal drug therapy. Additionally, we discussed the positive antacid fluorescence and antacid 2 test results with the pathologist. Subsequently, the pathologist examined the pathological sections and performed staining of the same tissue again, which revealed negative antacid fluorescence results, and no acid-fast bacteria were observed. Considering the treatment course and the mNGS results, we are sure that the original results were false positives and considered the samples to have been contaminated. Due to the high incidence of TB in China and the fact that the respiratory pathology center of our hospital receives and processes a large number of clinical specimens from patients with complex infections that show a high acid-fast bacilli positivity rate, we speculate that the most likely source of the contamination was from the water bath for the paraffin sections.

Finally, some insights from our case are as follows: (1) the importance of symptoms at follow-up, follow-up chest CT, and efficacy and safety assessments, should be emphasized; and (2) the importance of molecular techniques, such as mNGS, for the identification of rare fungi should be emphasized. This will improve the pathogen diagnosis rate when combined with other clinical microbiological tests.

## Supplementary Information

**Additional file 1: Figure S1.** Heatmaps of ANIb scores were constructed for 118 unique sequences of *Exophiala dermatitidis* from the patient and 12 *Exophiala* genomic sequences downloaded from the NCBI. Cells in the heatmap corresponding to a 95% or higher score (and therefore the same species) are colored in red. Orange cells correspond to ANIb scores of 95% or less, indicating that the corresponding organisms do not belong to the same species. Hierarchical clustering of the data in two dimensions is represented by dendrograms constructed by simple linkage of the ANIb score.

## Data Availability

The data that support the findings of the current study are available from the corresponding author upon reasonable request.

## Abbreviations

mNGS: Metagenomic next-generation sequencing
LRTI: Lower respiratory tract infection
TB: Tuberculosis
IGRA: Interferon gamma release assay
BALF: Bronchoalveolar lavage fluid
GM: Galactomannan
BG: (1 → 3)-β-D-glucan
ALT: Alanine aminotransferase
CF: Cystic fibrosis
MALDI-TOF: Matrix-assisted laser desorption ionization time-of-flight
EORTC/MSGERC: European Organization for Research and Treatment of Cancer and the Mycoses Study Group Education and Research Consortium
ESCMID: European Society of Clinical Microbiology and Infectious Diseases
ECMM: European Confederation of Medical Mycology
