# Supplementary figures and images for: The convoluted process of diagnosing pulmonary mycosis caused by Exophiala dermatitidis: a case report
Source: BMC Infect Dis. 2022 May 4;22:433. doi: 10.1186/s12879-022-07399-y (PMC9069750; doi:10.1186/s12879-022-07399-y)

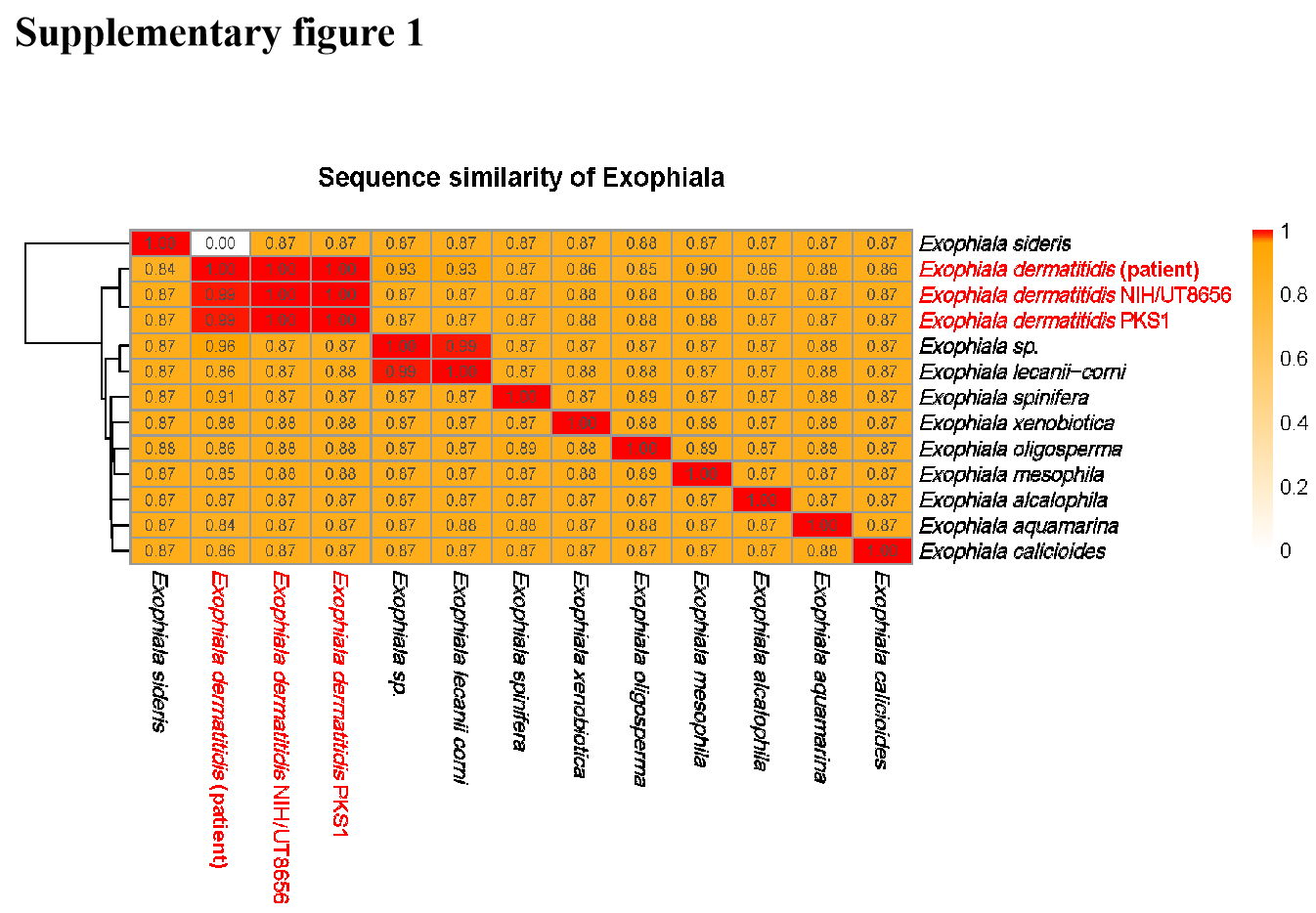

Supplement: Supplementary file 1 — Additional file 1: Figure S1. Heatmaps of ANIb scores were constructed for 118 unique sequences of Exophiala dermatitidis from the patient and 12 Exophiala genomic sequences downloaded from the NCBI. Cells in the heatmap corresponding to a 95% or higher score (and therefore the same species) are colored in red. Orange cells correspond to ANIb scores of 95% or less, indicating that the corresponding organisms do not belong to the same species. Hierarchical clustering of the data in two dimensions is represented by dendrograms constructed by simple linkage of the ANIb score. [file 12879_2022_7399_MOESM1_ESM.tif]
